# Supplementary material for: Simple within-stride changes in treadmill speed can drive selective changes in human gait symmetry
Source: PLoS One. 2023 Oct 26;18(10):e0287568. doi: 10.1371/journal.pone.0287568 (PMC10602355; doi:10.1371/journal.pone.0287568)
Supplement: Attachment — Submitted filename: PLOS One PONE-D-22-20229 Review.docx [file pone.0287568.s002.docx]

Review manuscript PLOS One

General Comments:

This manuscript describes a novel way to manipulate asymmetry via changing speeds on a treadmill within a stride. There were two main experiments, one where the speed changed based on the individuals propulsive force, and another where the speed changed during different parts of a gait cycle and a metronome was used to help the individual walk appropriately. I think the clinical applications of this work will be a great addition to the field and this study in healthy individuals shows promising results for this particular task. My primary concerns are in the statistical analysis and the role the metronome plays in influencing walking.

Major comments:

1. Even though these are healthy adults, did you do any tests to determine if these individuals walked symmetrically or asymmetrically to begin with? It seems most of the analysis was done during the period where the treadmill changed. I think the study would benefit from including a comparison to baseline walking, to see what changes the system is actually producing compared to how they typically walk. If all the analyses are within a condition comparing right and left, they do not test if that is how someone always walked or if the change in treadmill speed caused the asymmetry change.
2. While I agree that the metronome was a good way to get individuals to alter their walking pattern so the speeds could change at different portions of the gait cycle, I am curious if the authors think the metronome has any negative effects on the participant’s gait. For example, research has shown that walking to a metronome can alter individual’s gait variability. Therefore, are some of the changes in results in experiment 2 due to the metronome changing their natural gait or solely due to the treadmill speed?
3. Why did you choose a paradigm where the belts changed between 2 speeds instead of looking at an adaptive speed treadmill? Are there particular benefits of your approach versus the adaptive speed treadmill literature? While I think your approach is novel and has a lot of benefit, it may be worthwhile to further elaborate on the benefit of this versus the adaptive speed treadmill since there has been a variety of studies recently focused on that. This may be worth to touch on in the discussion.
4. I think it would be of more benefit to readers and help the reproducibility of the experiment if the statistics were presented in the text and in figures/tables for the actual manuscript and not just in the supplementary tables and figures.
5. Could you alter your presentation of your results to better match your statistical tests. For example, in the table, it seems that time may not be significant for many variables, but most of the text just emphasizes left versus right. I think it would be beneficial to the reader if both significant and insignificant results were presented.
6. Please reorganize your statistical tables. Based on what you had written in your methods, it seems you ran a separate ANOVA for each dependent variable (step length, step time, etc). However the way you present them by grouping by factor is confusing. I would have a table for each statistical test run and present it in the results.

Minor comments:

7. I would suggest presenting more of your statistical results (p values, etc) in the abstract.
8. Lines 63-65 state that: “Moreover, we expected that this would drive predictable, asymmetric changes in kinematic, kinetic, and spatiotemporal gait parameters that would depend on the timing of the speed change within the gait cycle.” Based on this hypothesis, it seems that the different timing of the speed changes would be compared to see if they presented differences. However, it did not seem the statistical analysis were set up that way. I would suggest to better align the hypotheses with the statistical approach.
9. How was the sample size determined? Did you do an apriori power analysis?
10. Line 104 mentions the treadmill acceleration was set to 6.0 m/s^2^. Did you consider looking at any mediolateral gait variables? I am curious if the fast acceleration influences stability. I think this would be important, especially for the clinical population application.
11. To clarify, for experiment 2, did the speeds change always at the same frequency for all four conditions, but it was just when the metronome occurred (signifying when to step) that changed?
12. For the statistical analysis, is there a certain reason why asymmetry or an asymmetry index was not used, and instead right vs left was compared? Calculating asymmetry may simplify some of the results and could directly target how asymmetry changed from normal walking to the controller conditions.
13. In the results, the time series plots are referenced when discussing the results of the statistical tests. I assume the first 30 or last 30 (the values from the bar plots) were used for the statistics. Therefore, I would suggest to update the reference figure to the bar plot or the statistical tables.
14. Did you have some way of seeing if individuals kept with the metronome?
15. For the beginning of each results section under experiment 2, it seems that some information would be better suited for the methods. For example in lines 236-240: “The Slow trial was designed to be the inverse of the Fast trial – here, participants walked with the open-loop controller activated and the treadmill moved slow over the first and last ~25% of the gait cycle and fast over the middle ~50% of the gait cycle (Figure 5A) – to test the robustness of our findings in the Fast trial (i.e., we expected the results to be opposite those observed in the Fast trial).” This describes what was done in the trial and doesn’t give any specific results. Therefore I would suggest to move these explanations to the methods.
