# Supplementary material for: Simple within-stride changes in treadmill speed can drive selective changes in human gait symmetry
Source: PLoS One. 2023 Oct 26;18(10):e0287568. doi: 10.1371/journal.pone.0287568 (PMC10602355; doi:10.1371/journal.pone.0287568)
Supplement: Attachment — Submitted filename: Response to Reviewers.docx [file pone.0287568.s004.docx]

Reviewer #2: Thanks to the authors for taking the time to address all comments. I have one further suggestion, and that is to include the specifics of your power analysis in the participants section of the paper.

**We thank the reviewer for their additional time reviewing this work. We added the details of the power analysis as suggested (lines 169-175 of the clean manuscript document).**
